# Assessing Cardiovascular Risk in Geriatric Patients Without Atherosclerotic Cardiovascular Disease

**DOI:** 10.3390/jcm13237133

**Published:** 2024-11-25

**Authors:** Witold Żurański, Justyna Nowak, Aleksander Danikiewicz, Barbara Zubelewicz-Szkodzińska, Bartosz Hudzik

**Affiliations:** 1 Third Department of Cardiology, Silesian Center for Heart Disease, Faculty of Medical Sciences in Zabrze, Medical University of Silesia, 40-055 Katowice, Poland; zuranskiwitold@gmail.com (W.Ż.); bhudzik@sum.edu.pl (B.H.); 2 Department of Cardiovascular Disease Prevention, Department of Metabolic Disease Prevention, Faculty of Public Health in Bytom, Medical University of Silesia, 40-055 Katowice, Poland; 3 Department of Nutrition-Related Disease Prevention, Department of Metabolic Disease Prevention, Faculty of Public Health in Bytom, Medical University of Silesia, 40-055 Katowice, Poland; adanikiewicz@sum.edu.pl (A.D.); bzubelewicz-szkodzinska@sum.edu.pl (B.Z.-S.); 4 Department of Endocrinology, District Hospital, 41-940 Piekary Slaskie, Poland

**Keywords:** ASCVD, frailty, multimorbidity, older people, SCORE2-OP

## Abstract

**Introduction:** Age is a major risk factor that affects the likelihood of developing atherosclerotic cardiovascular disease (ASCVD). The anticipated 10-year ASCVD risk for nearly all individuals aged 70 years and older surpasses conventional risk thresholds. When considering treatment for risk factors, it is important to take into account ASCVD risk modifiers, such as malnutrition, polypharmacy, and comorbidities. **Objectives:** The aim of this study was to estimate ASCVD risk in apparently healthy (without established ASCVD) elderly persons. We also evaluated several biochemical and clinical indicators to better characterize the studied population. **Patients and methods**: A total of 253 elderly individuals aged 70 years and older, who were apparently healthy and did not have established atherosclerotic cardiovascular disease (ASCVD), were enrolled in the study. The Systemic Coronary Risk Estimation 2-Older Persons (SCORE2-OP) model was utilized to assess their 10-year risk of developing ASCVD. **Results:** Among the 253 participants, 41 (16.2%) were classified as high risk, while 212 (83.8%) were categorized as very high risk. No individuals had a low ASCVD risk (defined as less than 7.5%). The median 10-year risk of developing ASCVD for the study group was 23% (ranging from 17% to 32%). The number of individuals identified as very high risk increased significantly with age, with nearly all participants aged 75 years and older being considered very high risk. An age of 75 years or older is associated with a very high risk for ASCVD, supported by a C-statistic of 0.92, which reflects a positive predictive value (PPV) of 99% and a negative predictive value (NPV) of 52% (*p* < 0.001). **Conclusions:** Elderly individuals without established ASCVD constitute a varied group. The majority were identified as being at very high risk for ASCVD. Age and hypertension were the primary factors contributing to this risk. Furthermore, modifiers of ASCVD risk, including malnutrition, polypharmacy, and multimorbidity, were commonly observed.

## 1. Introduction

With the development of industrialized countries and the improvement in economic conditions and medical advances, a significantly faster rate of population aging has recently been observed. Globally, in 2020, the number of people aged 60 years or older surpassed the number of children under the age of 5 years. Studies have indicated that by 2030, 1 in 6 people worldwide will be at least 60 years of age, and the number of elderly people will increase to 1.4 billion in 2030 [1]. In 2017, more than 20% of the population in Europe was at least 60 years of age. Furthermore, it is estimated that this percentage will increase to 35% in 2050 [2].

The aging process is a natural phenomenon that begins as early as during fetal life and lasts until death. It is caused by irreversible degeneration of cells and systems [3]. It is associated with several factors, including increased oxidative stress, inflammation, apoptosis, changes in the structure and function of the vessels and heart muscle, and a higher risk of developing other diseases, such as frailty syndrome, diabetes, or obesity [4]. Aging has its dynamics and includes physiological, psychological, sociological, and chronological changes [3,5].

Aging is a relative concept since chronological age does not always correspond to biological age. According to the traditional classification proposed by the World Health Organization (WHO), aging is classified as young-old (65–74 years of age), middle-old (75–84 years of age) and old-old (≥85 years of age) [6,7]. Chronological age does not reflect functional capacity, health, or productivity in the elderly population [2]. Old age is not a disease in itself.

However, age remains a strong, non-modifiable, and independent risk factor for cardiovascular diseases (CVDs) that are considered the leading cause of morbidity and mortality in the elderly [8]. Natural aging of the cardiovascular system and many comorbidities are associated with different management and treatment [9].

Primary prevention of CVDs is particularly effective in this group of patients as it prevents serious complications, such as strokes, myocardial infarcts and heart failure. Furthermore, it also improves the quality of life. However, cardiovascular risk assessment is difficult due to many aggravating factors, including frailty syndrome, multimorbidity, cognitive impairment, polypharmacy, or sarcopenia. Additionally, it is often based on an intuitive approach with a lack of appropriate tools supported by scientific evidence due to the small number of elderly people in scientific studies [8]. Moreover, different authors have often used arbitrary cut-offs of age to define the elderly population [2].

The Framingham Heart Study (FHS) identified classical CVD risk factors that increase the risk of CVDs in the general population [10]. These include age, gender, hypertension, diabetes, smoking, and obesity. The European Society of Cardiology (ESC) guidelines have recommended use of the SCORE charts to estimate an individual’s 10-year risk of fatal CVDs that include the following risk factors: age, gender, smoking, systolic blood pressure, and total cholesterol [11]. However, the assessment of cardiovascular risk in the elderly requires the consideration of several important aspects. In the past, the SCORE chart allowed risk estimation only in a narrow age range (i.e., between 40 and 65 years of age). In addition, the influence of classical risk factors decreases with age, and the risk of death from non-cardiovascular causes (“a competing risk”) plays an essential role in elderly patients. It leads to overestimating CVD risk and the potential treatment benefits. For this reason, in the new 2021 ESC guidelines on cardiovascular disease prevention, a new model known as SCORE2-Older Persons (SCORE2-OP), adjusted for the competing risk, was introduced to estimate an individual’s 10-year risk of fatal and non-fatal CVD events in apparently healthy people ≥70 years of age [12]. Because of difficulties in predicting risk in older people, utility of most available ASCVD risk scores is problematic, so there is limited capability to assess ASCVD risk in the elderly (70 years of age or older). Therefore, we set out to determine ASCVD risk in elderly people without established ASCVD. To better characterize the studied population, we also assessed several biochemical and clinical conditions associated with old age, such as multimorbidity, malnutrition, polypharmacy, and obesity, which can modify cardiovascular risk.

## 2. Patients and Methods

The analysis included 253 patients hospitalized in the Department of Geriatrics at the Medical Center in Piekary Śląskie, Poland. Data for this study were collected between 2022 and 2023. The inclusion criterion for the study was: apparently healthy subjects without ASCVD aged 70 years or older. The main exclusion criteria were medical conditions (ASCVD, diabetes, familial hypercholesterolemia, and/or renal failure) that prevented the use of SCORE2-OP. The anticipated 10-year risk of cardiovascular events and mortality for each patient was evaluated using the SCORE2-OP table for high-risk countries (Poland) [12].

The study had an all-comer design and it conformed to the Declaration of Helsinki. Informed consent for data analysis was obtained from the patients according to the Polish law on patients’ rights regarding data registration. The approval of a bioethics committee was not required for this study, considering that it was a retrospective analysis of an anonymized dataset.

Based on age, the study population was divided into four groups: Group 1 (*n* = 61) aged 70–74 years, Group 2 (*n* = 76) aged 75–79, Group 3 (*n* = 76) aged 80–84 and Group 4 (*n* = 43) aged 85–89 years. Medical records and hospital discharge summary reports of hospitalized patients were analyzed. A standard panel of laboratory tests was assessed for geriatric patients. The comorbidities, which included previously diagnosed and documented chronic conditions as well as newly diagnosed diseases during hospitalization, are shown in Table S1. We adopted the most commonly applied definition of multimorbidity, i.e., the coexistence of two or more chronic conditions in the same person [13]. In turn, polypharmacy was defined as using five or more medications [14]. The body mass index (BMI) was calculated by dividing weight in kilograms by height in meters squared. Patients were assigned to categories according to the classical division: underweight (BMI <18.5 kg/m^2^), normal weight (BMI range: 18.5 kg/m^2^–< 25 kg/m^2^), overweight (BMI: 25 kg/m^2^–<30 kg/m^2^) and obesity (BMI ≥ 30 kg/m^2^) [15]. Despite a lack of a clear definition of malnutrition, the 2015 consensus definition of the European Society for Clinical Nutrition and Metabolism was adopted [16].

### Statistical Analysis

Categorical variables are shown as absolute and relative frequencies (percentages). Continuous variables were initially tested for normality of data distribution by the Shapiro–Wilk test. The ANOVA test was used to analyze normal distribution data. The Kruskal–Wallis test was used to test non-normal distribution data. Normally distributed variables are expressed as mean ± standard deviation, whereas variables with a distribution other than normal are presented as median and interquartile range (25th–75th percentile). The relationship between age, SCORE2-OP, and other variables was evaluated by Spearman’s rank correlation coefficient. Multivariate logistic regression analysis was employed to evaluate odds ratios (OR) and 95% confidence intervals (95% CI) to evaluate the associations between age, lipid profile and laboratory findings, and very high cardiovascular risk. A stepwise analysis was performed, with *p* < 0.05 for inclusion and *p* > 0.2 for exclusion from the model in the overall population. A receiver operating characteristic (ROC) analysis was performed to assess the discrimination thresholds of age in identifying persons at a very high risk of ASCVD. A value of two-tailed *p* < 0.05 was considered significant. The statistical analyses were performed using Statistica13.0.

## 3. Results

Table 1 shows the clinical patient characteristics. All subjects were characterized by at least a high risk of ASCVD. In Groups 2, 3, and 4, patients with a very high risk of ASCVD were predominant (100% in Group 4). The prevalence of multimorbidity, polypharmacy, and malnutrition increased with age. In turn, body weight and BMI decreased. The highest number of obese patients was found in Group 1, while the highest systolic blood pressure values were observed in Group 4. Table 2 shows the results of laboratory tests. As regards the lipid profile, the concentrations of total cholesterol and triglycerides were the highest in Group 1. Similarly, the estimated glomerular filtration rate (eGFR) was the highest in patients aged 70–74 years and reached the lowest values in the oldest patients. Plasma concentrations of albumin and total protein did not differ significantly between the groups. The lowest values of the indicators of anemia were reported in Group 4. Table 3 shows the correlation between SCORE2-OP and various variables. Body weight, height, BMI, eGFR, and concentrations of plasma albumin and total protein were characterized by a weak negative correlation with SCORE2-OP. Similarly, Table 4 shows the correlation of age with other variables. Body weight, plasma glucose, triglyceride, LDL and non-HDL cholesterol levels, eGFR, plasma albumin and total protein concentrations, and anemia parameters, showed a weak negative correlation with age.

In turn, the number of diseases was associated with a moderate positive correlation. Table 5 shows the univariate and multivariate analysis of the variables and the correlation with very high cardiovascular risk. In the multivariate analysis, age increased the risk by 85% (OR 1.85), while HDL cholesterol concentration slightly reduced cardiovascular risk by 6% (OR 0.94). Table 6 shows the ROC curve analysis of several variables to predict very high cardiovascular risk. All variables were characterized by a high positive predictive value. However, only age determined a strong predictive value of very high cardiovascular risk (high sensitivity and specificity). HDL cholesterol levels, eGFR, MCV, and MCH were associated with a weak predictive value of very high cardiovascular risk.

## 4. Discussion

We assessed the cardiovascular risk in the population of elderly individuals without ASCVD. There are several crucial findings of this study. First, elderly patients were characterized by at least high cardiovascular risk, which increased with age and was highest among the oldest individuals. Second, age showed the strongest positive correlation with SCORE2-OP and a moderate positive correlation with the number of diseases compared to all variables. Third, SCORE2-OP showed a weak negative correlation with HDL cholesterol concentration as the only component of the lipid profile. Furthermore, HDL cholesterol concentration slightly reduced cardiovascular risk. Only age was associated with a strong predictive value of very high cardiovascular risk.

ASCVD remains a major cause of morbidity and mortality worldwide [12]. In recent decades, many studies have identified traditional ASCVD risk factors [17]. It has allowed the inclusion of appropriate ASCVD prevention, which has resulted in significantly lower rates of deaths from myocardial infarction and stroke [18]. However, many of these studies were conducted in middle-aged populations and the elderly subjects were excluded, which makes it impossible to reliably extrapolate these results to the population of elderly patients [19].

The association between traditional risk factors and CVDs becomes weaker with age [20]. The pathophysiological changes that cause CVDs in the elderly also trigger other conditions. Therefore, CVDs in the elderly tend to co-exist with other diseases [8]. Multimorbidity can occur at any age. However, the number and complexity of comorbidities increases with age [8]. Aging-related structural changes in large arteries decrease their compliance and elasticity, which results in isolated hypertension in clinical practice [9]. An increase in systolic blood pressure results in an increase in cardiac afterload and a decrease in coronary perfusion, which can lead to myocardial ischemia, acute coronary syndrome, and heart failure [4]. It is one of the instances in which chronic diseases are linked by the underlying pathophysiology or treatment-related problems [21,22]. Other conditions may be pathogenetically related to a lesser extent.

Hypertension is a very common cardiovascular risk factor in the elderly population. It increases with age, affecting over 60% of individuals aged 60 years and older [23]. According to Zieleniewicz P. et al. [23], the NOMED-AF study showed a high prevalence (over 80% for both sexes) and suboptimal control of hypertension in Poland’s elderly population. The prevalence of hypertension in our study ranged from 75.3 to 86.8% for both sexes and was similar to the cohort of patients in the NOMED-AF study for the Polish population. Such a high prevalence of hypertension in the elderly population points to a significant public health challenge, as hypertension is the dominant driver of cardiovascular risk in this group of patients, and its appropriate treatment may reduce not only the rate of cardiovascular events but also all-cause mortality [24].

Malnutrition is a highly prevalent condition in the elderly [25]. According to the multicenter studies, the prevalence of malnutrition is estimated at 23–60% of elderly patients, and approximately 22–28% are at risk of malnutrition [26]. In our cohort of studied patients, the prevalence of malnutrition was 19.7 to 34.9% of individuals and increased with age. During the shift into older years, the nutrition priorities change toward meeting increased nutrient needs with lower energy requirements (protein-energy malnutrition; PEM) [26], which leads to the loss of muscle mass, sarcopenia, and cachexia. Moreover, malnutrition induces chronic inflammation, which in turn promotes arteriosclerosis with intravascular disorders and calcification. Thus, malnutrition may accelerate arteriosclerosis and increase major adverse cardiovascular events (MACE) and kidney events [27]. As a result of the correlation between malnutrition and hypertension, the concept of malnutrition–inflammation–atherosclerosis (MIA) syndrome has recently been proposed [27]. All the components of MIA syndrome interact with each other, forming a vicious cycle. Given that malnutrition is not a typical cardiovascular risk factor in older persons and the control of hypertension in treated elderly individuals is poor (in the Polish population it is 31–38% in the Polsenior study and 40–45% in NOMED-AF), it should be emphasized that early detection and treatment of malnutrition and enhanced strategies in hypertension care point to a significant public health challenge. Malnutrition and hypertension are modifiable factors, so early detection and intensive treatment without unnecessary delays can improve patients’ prognosis and reduce the risk of adverse cardiovascular events [23,27,28,29,30].

Obesity is considered to be one of the important cardiovascular risk factors, but it was not included in the SCORE2-OP algorithm. It is a component of metabolic syndrome and mostly determined by BMI [31]. According to Suwała et al. [32], BMI is a reliable predictor of an above-average increased cardiovascular risk (ICVR) [32]. Additionally, a new optimal cut-off value for BMI of 27.6 kg/m^2^ was proposed for both sexes [32]. In our study, patients in the younger age groups had a higher BMI value with a lower cardiovascular risk estimated by the SCORE2-OP algorithm. A novel BMI threshold of 27.6 kg/m^2^ increases cardiovascular risk by 3.3–5.3 times [32]. This suggests that patients in the younger age groups may have an underestimated cardiovascular risk based on the SCORE2-OP algorithm, which contributes to therapeutic inertia.

Because of the uncertainties associated with estimating cardiovascular risk using the SCORE2-OP algorithm, Trier et al. [33] conducted a study to assess whether this risk estimation model is accurate and can guide decisions about preventive treatment in older persons. The overall ability to discriminate between high- and low-risk patients was poor, demonstrated by an AUC of 0.63 (95% CI 0.60–0.65), regardless of gender. However, in this study, the SCORE2-OP low-risk model was used in people aged 70–79 years in the UK population cohort from the EPIC-Norfolk study from 1993–1997. In our study, the SCORE2-OP high-risk model was used (Polish population) in patients aged 70–89 years, and data for this study were collected between 2022 and 2023. There were also differences in cohort size: 3,113 vs. 253, respectively. Despite these differences between the two studies, it is important to consider individual risk factors, other risk factors, and risk modifiers not included in this model during shared decision-making on whether to initiate or intensify treatment [33].

Polypharmacy is another risk modifier resulting in adverse health outcomes [34,35]. The overall prevalence of polypharmacy in the elderly population worldwide is nearly 40% and is significantly higher in elderly individuals aged 70 or older [36]. The study cohort had a high prevalence of polypharmacy, especially in the oldest group, which is consistent with observations in other studies [36]. Polypharmacy is an independent risk factor for major cardiovascular events, cardiovascular disease-specific, and all-cause mortality in older people (aged ≥ 65 years) [35]. This highlights the need to decrease unnecessary polypharmacy, optimize care of older people, and reduce drug-related issues [34,35].

Apart from polypharmacy, frailty has the potential to modify the risk of adverse outcomes [37]. Frailty syndrome is a state of increased vulnerability to physiological stressors and reduced homeostatic reserve [38,39]. According to Kojima et al. [40], people with frailty syndrome are 2–3-fold more likely to experience disability, deterioration of overall fitness, and falls. Additionally, being a marker of increased risk of adverse effects in CVD, frailty syndrome increases morbidity and mortality by at least 2-fold [34,35,37,38]. Frailty syndrome may suggest implementing alternative and less burdensome treatment when the risk of management is too high.

It is worth noting that changes in autonomic balance can be a marker for frailty in older adults. [41]. Changes in cardiac autonomic modulation may result in increased sympathetic modulation and a higher cardiovascular risk in this population. Using proper tools to monitor heart rate variability (HRV) (modulated by the sympathetic and parasympathetic systems) would be ideal for the older adult population and can be a strategy for prevention of frailty and CVD. [41].

All of the above conditions can lead to functional decline, which is associated with a poorer prognosis, reduced quality of life, loss of independence, and increased hospitalization rates [39]. In addition, management based on guidelines and recommendations resulting in prescribing many medications can lead to limited dose escalation, reduction in medication use, and poor compliance [40].

Many of the above conditions are not included in numerous predictive models of cardiovascular risk. In the past, the ESC recommended the use of SCORE to assess cardiovascular risk. However, its application was not possible for people over 65 years of age [11]. Alternative scales used to evaluate cardiovascular risk in the elderly include QRISK3 for patients aged 25–84 years [42], CVDPoRT for patients aged 20–105 years [43], the Pooled Cohort Equations for patients aged 40–79 years [44], and revised WHO CVD risk estimation charts for patients aged 40–74 years [45]. Furthermore, none of the scales considers imaging studies or plasma markers in assessing cardiovascular risk. According to Mortensen et al. [46], CAC = 0, CAC ≤ 10, low galectin-3 and no carotid plaque could serve as negative markers of cardiovascular events in elderly patients (HR 0.35, 0.27, 0.31, 0.55, respectively). According to Kasim et al. [47], many similar predictive models have shown a tendency to overestimate the 10-year cardiovascular risk by a significant amount, ranging from 3% to 1430% [47]. Therefore, the search for a perfect risk assessment model is futile and user-friendly models would work best in everyday clinical practice.

## 5. Conclusions

In conclusion, age and high blood pressure are the main cardiovascular risk factors in the elderly without established ASCVD. Despite the differences between age groups, all individuals were characterized by at least high cardiovascular risk. In this work, as in other studies and meta-analyses, multimorbidity, polypharmacy, and malnutrition are highly prevalent in the elderly and are considered as ASCVD risk modifiers. Despite the existence of special scales to assess the cardiovascular risk in the elderly, many of these conditions are not included. Knowledge of the diseases of old age and clinical vigilance in their diagnosis are crucial. Such vigilance can significantly affect the treatment process and prognosis in this heterogeneous group of patients.

## 6. Limitation of the Study

Some aspects of our study warrant consideration. First, the study has an all-comer design, and we did not consider follow-up. Second, the entire age range of SCORE2-OP was evaluated (70–89 years). However, the traditional definition of old age according to the WHO is age ≥ 65 years. Therefore, the young-old group (65–69 years) was not included in our cohort. Third, a significant drawback of this study is the relatively low number of participants—253. Another concern may arise from the geographically homogeneous sample (all patients came from Silesia, a specific region in Poland). However, the prevalence of ASCVD risk factors is similar compared to populations studied in other regions of Poland.

## Figures and Tables

**Table 1:** Baseline characteristics.

Variable	Group 1 70–74 Years (*n* = 61)	Group 2 75–79 Years (*n* = 73)	Group 3 80–84 Years (*n* = 76)	Group 4 85–89 Years (*n* = 43)	*p*-Value
10-year ASCVD, median (IQR)	14 (12–17)	20 (17–23)	30 (26–33)	42 (35–50)	<0.001
10-year ASCVD risk categories, *n* (%)					<0.001
Low risk (<7.5%)	0 (0.0)	0 (0.0)	0 (0.0)	0 (0.0)	
High risk (7.5–14.9%)	34 (55.7)	6 (8.2)	1 (1.3)	0 (0.0)	
Very high risk (≥15%)	27 (44.3)	67 (91.2)	75 (98.7)	43 (100)	
Age, years, mean (SD)	72 ± 1	77 ± 1	82 ± 1	87 ± 2	<0.001
Hypertension, *n* (%)	51 (83.6)	55 (75.3)	66 (86.8)	35 (81.4)	0.3
Hypercholesterolemia, *n* (%)	14 (29.5)	19 (26.0%	13 (17.1)	9 (20.9)	0.6
Multimorbidity, *n* (%)	40 (65.6)	56 (76.7)	63 (82.9)	38 (88.4)	0.02
Polypharmacy, *n* (%)	38 (62.3)	50 (68.5)	60 (78.9)	36 (83.7)	0.02
Malnutrition, *n* (%)	12 (19.7)	18 (24.7)	25 (32.9)	15 (34.9)	0.02
Smoking, *n* (%)	9 (15.3)	4 (5.7)	5 (6.9)	2 (4.8)	0.2
Weight, kg, mean (SD)	74 ± 15	70 ± 16	65 ± 12	64 ± 11	<0.001
Systolic blood pressure, mmHg, mean (SD)	138 ± 12	145 ± 9	150 ± 8	155 ± 8	0.03
BMI, kg/m^2^, mean (SD)	29 ± 5	28 ± 6	27 ± 4	26 ± 4	0.001
BMI categories, *n* (%)					0.04
Underweight	1 (1.6)	3 (4.1)	1 (1.3)	0 (0.0)	
Normal weight	14 (23.0)	16 (21.9)	30 (39.5)	14 (32.6)	
Overweight	19 (31.1)	32 (43.8)	30 (39.5)	19 (44.2)	
Obesity	27 (44.3)	22 (30.1)	15 (19.7)	10 (23.2)	

Abbreviations: ASCVD, atherosclerotic cardiovascular disease; BMI, body mass index; IQR, interquartile range; SD, standard deviation.

**Table 2:** Laboratory findings.

Variable	Group 1 70–74 Years (*n* = 61)	Group 2 75–79 Years (*n* = 73)	Group 3 80–84 Years (*n* = 76)	Group 4 85–89 Years (*n* = 43)	*p*-Value
TSH, µIU/mL, median (IQR)	1.15 (0.85–1.71)	1.15 (0.59–2.04)	1.18 (0.66–1.7)	1.12 (0.74–1.89)	0.99
Glucose, mg/dL, median (IQR)	104.5 (91.5–129)	97 (90–114)	100 (87–105)	95 (87–105)	0.08
Na, mmol/L, median (IQR)	141 (139.5–143)	140 (138–142)	141 (139–143)	140 (138–142)	0.12
K, mmol/L, median (IQR)	4.5 (4.2–4.7)	4.3 (4.0–4.6)	4.3 (4.0–4.7)	4.4 (4.1–4.6)	0.11
Total cholesterol, mg/dL, median (IQR)	187 (171–216)	174 (145–213)	173.5 (155–208.5)	169 (142–206)	0.05
HDL cholesterol, mg/dL, median (IQR)	54 (42–67)	52 (41–63)	52 (44–65.5)	55 (43–64)	0.91
LDL cholesterol, mg/dL, median (IQR)	109.2 (93–132.2)	105.6 (80.8–137.4)	101.4 (79.5–128.5)	96 (70.6–130.4)	0.19
Triglycerides, mg/dL, median (IQR)	117 (94–156)	98 (79–126)	101 (80–130)	96 (78–110)	<0.01
Glomerular filtration, mL/min/1.73 m^2^, median (IQR)	78.5 (67.8–91)	76 (55–84)	73 (59–83)	66 (41–81)	<0.01
Creatinine, mg/dL, median (IQR)	0.81 (0.7–0.9)	0.87 (0.72–1.04)	0.87 (0.69–1.07)	0.93 (0.74–1.21)	0.08
Aspartate aminotransferase, U/liter, median (IQR)	19 (16–23)	18 (14–22)	17 (13–21.5)	18 (16–24)	0.26
Alanine aminotransferase, U/liter, median (IQR)	17 (14–25)	14 (12–20)	15.5 (11–20)	15 (11–18)	0.03
Serum albumin, g/L, median (IQR)	36 (31.5–39.5)	35 (31–38)	34 (32–37)	33 (3.7–36)	0.02
Total protein, g/L, median (IQR)	67 (64–69.3)	65.5 (62.5–67)	60.6 (58–64.7)	65.8 (57.8–67.1)	<0.01
White cell count, 10³/µL, median (IQR)	6.6 (5.1–8.3)	6.2 (5.0–7.6)	5.8 (5.05–7.25)	6.6 (5.3–7.8)	0.41
Red cell count, 10⁶/µL, median (IQR)	4,38 (4.2–4.77)	4,24 (3.81–4.52)	4,13 (3.85–4.5)	4,11 (3.85–4.42)	0.02
Hemoglobin, g/dL, median (IQR)	12.9 (12.1–13.9)	12.5 (11.1–13.6)	12.6 (11.6–13.25)	12.1 (11.0–13.1)	0.02
Hematocrit, %, median (IQR)	39.5 (37.6–42.3)	38.4 (34.1–41.5)	37.55 (35.55–41.05)	37.3 (34.6–40.8)	0.02
Platelet count, 10³/µL, median (IQR)	210 (173–269)	220 (179–267)	212.5 (165–253)	211 (160–242)	0.64

Abbreviations: IQR, interquartile range.

**Table 3:** The correlation between SCORE2-OP and other variables.

**SCORE2-OP**	**Variable**	***n***	**R**	***p***
	Weight, kg	253	−0.27	<0.001
	Height, cm	253	−0.18	0.004
	BMI, kg/m^2^	253	−0.19	0.003
	TSH (µIU/L)	243	0.01	0.85
	Serum glucose, mg/dL	252	−0.07	0.24
	Serum sodium, mmol/L	253	−0.02	0.71
	Serum potassium, mmol/L	253	−0.03	0.59
	Triglycerides, mg/dL	252	−0.02	0.72
	Vitamin B₁₂	146	0.07	0.40
	Glomerular filtration, mL/min	219	−0.23	<0.001
	Serum creatinine, mg/dl	252	0.12	0.06
	Aspartate aminotransferase, U/L	253	−0.01	0.9
	Alanine aminotransferase, U/L	253	−0.08	0.19
	Bilirubin, mg/dL	249	0.05	0.46
	Serum albumin, g/dL	124	−0.2	0.03
	Total protein, g/dL	144	−0.17	0.04
	Number of diseases	253	0.18	0.04

**Table 4:** The correlation between age and other variables.

**Age, Years**	**Variable**	***n***	**R**	***p***
	Weight, kg	253	−0.29	<0.001
	Height, cm	253	−0.20	<0.01
	BMI, kg/m^2^	253	−0.20	<0.01
	TSH (µIU/L)	243	−0.01	0.88
	Serum glucose, mg/dL	252	−0.14	0.02
	Serum sodium, mmol/L	253	−0.08	0.18
	Serum potassium, mmol/L	253	−0.09	0.14
	Total cholesterol, mg/dL	253	−0.14	0.02
	HDL cholesterol, mg/dL	253	0.01	0.87
	LDL cholesterol, mg/dL	253	−0.13	0.03
	Triglycerides, mg/dL	252	−0.19	<0.01
	Non-HDL cholesterol, mg/dL	253	−0.15	0.01
	Glomerular filtration, ml/min	219	-0.24	<0.001
	Serum creatinine, mg/dl	252	0.15	0.02
	Aspartate aminotransferase, U/L	253	−0.03	0.63
	Alanine aminotransferase, U/L	253	−0.12	0.06
	Bilirubin, mg/dl	249	0.08	0.20
	Serum albumin, g/dL	144	−0.20	0.01
	Total protein, g/dL	124	−0.29	<0.001
	White blood cells, 10^3^/µL	253	−0.02	0.68
	Hemoglobin, g/dL	253	−0.16	<0.001
	Red blood cells, 10^6^/µL	253	−0.20	<0.02
	Hematocrit, %	253	−0.16	0.01
	Platelet count, 10^3^/µL	253	−0.09	0.16
	Mean corpuscular volume, fl	250	0.10	0.10
	Mean corpuscular hemoglobin, pg	250	0.09	0.14
	Mean corpuscular hemoglobin concentration, g/dL	250	−0.03	0.65
	Lymphocytes	251	−0.06	0.30
	Monocytes	238	0.06	0.37
	Eosinophils	34	−0.14	0.42
	Number of diseases	253	0.36	0.01

**Table 5:** Univariate and multivariate logistic regression analysis to evaluate the associations between age, lipid profile, and laboratory findings, and very high cardiovascular risk.

Variable	Univariate			Multivariate		
	OR	95% CI	*p*	OR	95% CI	*p*
Age	1.71	1.44–2.04	<0.001	1.85	1.51–2.26	<0.001
HDL cholesterol	0.98	0.96–0.99	<0.01	0.94	0.91–0.98	<0.001
eGFR	0.98	0.96–0.99	<0.01	-	-	-
MCV	1.04	1.00–1.08	0.05	-	-	-
MCHC	1.13	1.00–1.29	0.05	-	-	-

Abbreviations: eGFR, estimated glomerular filtration rate; MCHC, mean corpuscular hemoglobin concentration; MCV, mean corpuscular volume.

**Table 6:** Receiver-operating characteristic (ROC) curves identifying discrimination thresholds of age, weight, BMI, and laboratory findings for determining very high cardiovascular risk.

Variable	Cut-Off	AUC	95% CI	Sensitivity (%)	Specificity (%)	PPV (%)	NPV (%)	*p*
Age	>75	0.92	0.88–0.95	83	97	99	52	<0.001
BMI	<28	0.55	0.48–0.61	-	-	-	-	0.35
eGFR	<55	0.63	0.56–0.69	27	95	96	21	<0.01
Hemoglobin	<13	0.51	0.45–0.57	-	-	-	-	0.83
HDL cholesterol	<59	0.65	0.59–0.71	68	61	90	26	<0.001
Hematocrit	<35	0.53	0.47–0.59	-	-	-	-	0.5
LDL cholesterol	>90	0.54	0.48–0.61	-	-	-	-	0.35
MCH	<30.5	0.62	0.56–0.68	34	88	93	21	<0.01
MCHC	<32.6	0.57	0.49–0.63					0.14
MCV	<90	0.62	0.55–0.68	61	61	89	23	0.01
Non-HDL cholesterol	>108	0.52	0.46–0.58	-	-	-	-	0.67
Platelet count	>251	0.56	0.49–0.63	-	-	-	-	0.14
RBC	<4.1	0.55	0.49–0.61	-	-	-	-	0.24
Serum albumin	<39	0.52	0.44–0.60	-	-	-	-	0.78
Serum glucose	>120	0.5	0.44–0.57	-	-	-	-	0.93
Total cholesterol	>175	0.57	0.48–0.63	-	-	-	-	0.18
Triglycerides	>100	0.53	0.47–0.59	-	-	-	-	0.5
WBC	>4.5	0.5	0.44–0.56	-	-	-	-	0.97
Weight	>71	0.54	0.47–0.60	-	-	-	-	0.47

Abbreviations: BMI, body mass index; eGFR, estimated glomerular filtration rate; HDL cholesterol, High-Density Lipoprotein cholesterol; LDL cholesterol, Low-Density Lipoprotein cholesterol; MCH, mean corpuscular hemoglobin; MCHC, mean corpuscular hemoglobin concentration; MCV, mean corpuscular volume; RBC, red blood cells; WBC, white blood cells.

## Data Availability

The data presented in this study are available on request from the corresponding author.

## Supplementary Materials

The following supporting information can be downloaded at: https://www.mdpi.com/article/10.3390/jcm13237133/s1, Table S1: The table groups the reasons for hospitalization.
