# Supplementary material for: Assessing Cardiovascular Risk in Geriatric Patients Without Atherosclerotic Cardiovascular Disease
Source: J Clin Med. 2024 Nov 25;13(23):7133. doi: 10.3390/jcm13237133 (PMC11641976; doi:10.3390/jcm13237133)
Supplement: Supplementary file 1 [file jcm-13-07133-s001.zip › jcm-3231623-supplementary.pdf]

Table S1. The table groups the reasons for hospitalization.

**Respiratory system diseases**	• Chronic obstructive pulmonary disease (COPD<br>• Asthma<br>• Obstructive sleep apnea (OSA)<br>• Interstitial lung disease (ILD)
**Digestive system diseases**	• Gastroesophageal reflux disease (GERD)<br>• Hiatal hernia<br>• Irritable bowel syndrome (IBS)<br>• Diverticular disease<br>• Colon polyps and cancer<br>• Hemorrhoids
**Urinary tract diseases**	• Urinary tract infection (UTI)<br>• Pyelonephritis<br>• Nephrolithiasis<br>• Urinary incontinence<br>• Urological neoplasia
**Rheumatological disease**	• Rheumatoid arthritis<br>• Systemic lupus erythematosus (SLE)<br>• Systemic sclerosis<br>• Dermatomyositis<br>• Sjögren syndrome<br>• Polymyalgia rheumatica (PMR)
**Neurological disorders**	• Alzhaimer’s Disease<br>• Parkinson's Disease<br>• Subdural haematoma<br>• Headache<br>• Vertigo
**Skin disorders**	• Lupus<br>• Psioriasis<br>• Skin cancer<br>• Vitiligo

**Oncology**	• Prostate cancer<br>• Bladder cancer<br>• Lung cancer<br>• Colon cancer<br>• Acute myeloid leukemia (AML)<br>• Chronic lymphocytic leukemia (CLL)<br>• Chronic myeloid leukemia (CML)
